# Cerebral oxygenation in health and disease

**DOI:** 10.3389/fphys.2014.00458

**Published:** 2014-11-24

**Authors:** Patrice Brassard, Philip N. Ainslie, Niels H. Secher

**Affiliations:** ^1^Department of Kinesiology, Faculty of Medicine, Université Laval, QuébecQC, Canada; ^2^Centre for Heart, Lung and Vascular Health, School of Health and Exercise Sciences, University of British Columbia-OkanaganKelowna, BC, Canada; ^3^The Copenhagen Muscle Research Center, Department of Anesthesia, University of CopenhagenCopenhagen, Denmark

**Keywords:** cerebral oxygenation, cerebral blood flow, near-infrared spectroscopy

Monitoring cerebral blood flow (CBF) and oxygenation has implications for both clinical practice and research interests; e.g., to provide insight into functional neurovascular coupling, to better understand orthostatic hypotension, and to evaluate the influence of vasopressors on cerebral oxygenation during anesthesia and/or surgery. These topics, and others, are addressed in this e-book by presenting original research, reviews, and opinion papers covering new, exciting but also controversial issues related to cerebral oxygenation in health and disease as evaluated by near-infrared spectroscopy (NIRS).

There is interest in the impact of vasopressors on the NIRS-determined frontal lobe oxygenation (S_c_O_2_). For example, a reduction in S_c_O_2_ is reported with use of phenylephrine and noradrenaline at rest in healthy subjects, during anesthesia in non-cardiac and cardiac patients and during cardiopulmonary bypass in diabetics. However, possible extracranial contamination of the NIRS signal, especially with the utilization of vasopressors, challenges these conclusions. Keeping this limitation in mind, Foss et al. (2014) explored the influence of phenylephrine and ephedrine, on S_c_O_2_ during cesarean section with spinal anesthesia. Both vasopressors were effective at maintaining mean arterial pressure (MAP). Still, phenylephrine was the agent associated with a reduction in S_c_O_2_. In addition, Kitchen et al. (2014) studied the effect of calcium chloride compared to α- and β-adrenergic receptor agonists (ephedrine, phenylephrine, adrenaline, or noradrenaline) following anesthesia-induced hypotension in patients scheduled for major abdominal surgery. This case series suggested that S_c_O_2_ was preserved in patients who received calcium chloride, as well as β-adrenergic receptor agonists, but slightly reduced (2%) in those who received α-adrenergic drugs.

Also, ventilation, O_2_ supplementation and body position have the potential to affect NIRS-derived S_c_O_2_ during surgery. Larsen et al. (2014) explored whether induction of anesthesia in the reclining, compared to sitting beach-chair position, secures cerebrovascular hemodynamics, including S_c_O_2_. S_c_O_2_ was found to be higher, combined with more stable hemodynamics, characterized by a reduced utilization of ephedrine, following induction of anesthesia in the reclining beach-chair vs. the sitting position. In their retrospective analyses of patients undergoing liver transplantation, Sørensen et al. (2014) report that S_c_O_2_ changes during surgery were closely related to those in end-tidal carbon dioxide tension. In order to ensure stability in S_c_O_2_ during the different phases of a liver transplantation, a varying ventilatory strategy may be needed to reduce the incidence of postoperative complications. Rokamp et al. (2014a) examined whether O_2_ supplementation could maintain S_c_O_2_ and skeletal muscle oxygenation in vascular surgical patients. These authors conclude that O_2_ supplementation indeed elevates S_c_O_2_ and skeletal muscle oxygenation in these patients during surgery but does not seem to sufficiently prevent a critical reduction in S_c_O_2_. Nielsen (2014) reviewed the impact of different surgeries on S_c_O_2_. His report indicates that the impact of non-cardiac surgery on S_c_O_2_ is highly variable and in some types of surgery, cerebral desaturation may be related to postoperative cognitive dysfunction.

Arterial pressure influences CBF. However, the role of arterial pressure variability on clinical outcome is not clear. Bronzwaer et al. (2014) explored the relationship between arterial pressure variations, stroke volume index and regional cerebral perfusion during transient central blood volume depletion and repletion in healthy volunteers and found that middle cerebral artery flow velocity (MCA Vmean) is related linearly to arterial pressure variability in subjects under these conditions. In their review, Rickards and Tzeng (2014) tried to reconcile two apparently discrepant views regarding variability in arterial pressure and CBF (negative vs. positive impact on clinical outcome), and suggest that the time scale of hemodynamic variability, that is short time variability vs. longer term fluctuations, may be the key to merge these divergent views.

To better understand the integrative components of cerebrovascular control, and thus oxygenation, during hyperthermia, Bain et al. (2014) discuss the mechanisms related to CBF and oxygenation changes during moderate to severe levels of hyperthermia. On the opposite spectrum, a reduction in cerebral temperature (hypothermia) may be important, for example to prevent cerebral ischemia during anesthesia or to improve neurological outcome and survival after cardiac arrest. Nybo et al. (2014) explored the impact of different means of brain cooling on cerebral temperature balance and oxygenation, namely intranasal cooling, percutaneous cooling of the carotid arteries and nasal ventilation.

Other physiological challenges influence CBF and oxygenation. Rokamp et al. (2014b) explored whether cholinergic vasodilatation is of importance for the elevation in regional CBF, measured by arterial spin labeling and blood O_2_ level dependent functional magnetic resonance imaging during a handgrip motor task and visual light stimulation in healthy subjects. By using blockade of acetylcholine receptors, known to abolish the exercise-induced increase in MCA Vmean, they observed that the elevation in regional CBF does not seem to be affected by glycopyrrolate. Also, since associations between arterial stiffness and cerebrovascular pulsatility have only been cross-sectional, and that resistance exercise increases arterial stiffness, Lefferts et al. (2014) explored whether increases in arterial stiffness induced by acute resistance exercise elevates CBF velocity pulsatility. While resistance exercise increased carotid artery stiffness and pressure pulsatility, this type of acute exercise did not affect CBF pulsatility. The impact of the Valsalva maneuver (VM) on different physiological functions is well documented but its influence on cerebrovascular function, including beat-to-beat measures of CBF velocity and oxygenation, have not been reported. Perry et al. (2014) studied the impact of 30 and 90% of subjects' maximal VM mouth pressure on MCA velocity and S_c_O_2_. They observed greater reductions in CBF velocity and oxygenation during phase II and III of the VM with the more intense maneuver. The latter was associated with a larger overshoot of CBF velocity and oxygenation after release of the strain (phase IV).

Aging influences cerebrovascular function. Flück et al. (2014) examined the relationship between CBF responses to a visual stimulus and a hypercapnic challenge with cerebral augmentation index, i.e., a measure of arterial stiffening. They conclude that MCA Vmean responses to both challenges were reduced with advancing age, with a parallel elevation in cerebrovascular stiffness. In their review, Tarumi and Zhang (2014) present the current evidence in regards to how vascular health affects the aging brain, and how improvements in vascular health, especially through regular aerobic exercise, may benefit cognitive function.

While different methodologies exist to monitor neural control of gait in humans, Perrey (2014) focused on the utilization of functional NIRS to perform brain imaging during walking, with emphasize on the sensitivity and pitfalls of this methodology in an opinion paper. Even if NIRS represents an interesting non-invasive tool to assess cerebral oxygenation, Grocott and Davie (2013) highlight the uncertainties, which could prevent this methodology from reaching its full potential. Finally, Sørensen et al. (2013) provide some insight to improve algorithms used in NIRS devices.

This research topic covers a wide range of exciting but at the same time unsettled issues related to cerebrovascular physiology with a focus on cerebral oxygenation. We hope that this e-book represents an opportunity to improve understanding in this field, as well as provide directions for further research.

## Conflict of interest statement

The authors declare that the research was conducted in the absence of any commercial or financial relationships that could be construed as a potential conflict of interest.

## References

[B1] Bain A. R. Morrison S. A. Ainslie P. N. (2014). Cerebral oxygenation and hyperthermia. Front. Physiol. 5:92. 10.3389/fphys.2014.00092 24624095 PMC3941303

[B2] Bronzwaer A. S. Stok W. J. Westerhof B. E. van Lieshout J. J. (2014). Arterial pressure variations as parameters of brain perfusion in response to central blood volume depletion and repletion. Front. Physiol. 5:157. 10.3389/fphys.2014.00157 24795652 PMC4006039

[B3] Flück D. Beaudin A. E. Steinback C. D. Kumarpillai G. Shobha N. McCreary C. R.. (2014). Effects of aging on the association between cerebrovascular responses to visual stimulation, hypercapnia and arterial stiffness. Front. Physiol. 5:49. 10.3389/fphys.2014.00049 24600398 PMC3928624

[B4] Foss V. T. Christensen R. Rokamp K. Z. Nissen P. Secher N. H. Nielsen H. B. (2014). Effect of phenylephrine vs. ephedrine on frontal lobe oxygenation during caesarean section with spinal anesthesia: an open label randomized controlled trial. Front. Physiol. 5:81. 10.3389/fphys.2014.00081 24624090 PMC3940064

[B5] Grocott H. P. Davie S. N. (2013). Future uncertainties in the development of clinical cerebral oximetry. Front. Physiol. 4:360. 10.3389/fphys.2013.00360 24385967 PMC3866380

[B6] Kitchen C. C. Nissen P. Secher N. H. Nielsen H. B. (2014). Preserved frontal lobe oxygenation following cacium chloride for treatment of anesthesia-induced hypotension. Front. Physiol. 5:407. 10.3389/fphys.2014.00407 25374543 PMC4205832

[B7] Larsen S. L. Lyngeraa T. S. Maschmann C. P. Van Lieshout J. J. Pott F. C. (2014). Cardiovascular consequence of reclining vs. sitting beach-chair body position for induction of anesthesia. Front. Physiol. 5:187. 10.3389/fphys.2014.00187 24904427 PMC4032912

[B8] Lefferts W. K. Augustine J. A. Heffernan K. S. (2014). Effect of acute resistance exercise on carotid artery stiffness and cerebral blood flow pulsatility. Front. Physiol. 5:101. 10.3389/fphys.2014.00101 24678301 PMC3958641

[B9] Nielsen H. B. (2014). Systematic review of near-infrared spectroscopy determined cerebral oxygenation during non-cardiac surgery. Front. Physiol. 5:93. 10.3389/fphys.2014.00093 24672486 PMC3955969

[B10] Nybo L. Wanscher M. Secher N. H. (2014). Influence of intranasal and carotid cooling on cerebral temperature balance and oxygenation. Front. Physiol. 5:79. 10.3389/fphys.2014.00079 24578693 PMC3936139

[B11] Perrey S. (2014). Possibilities for examining the neural control of gait in humans with fNIRS. Front. Physiol. 5:204. 10.3389/fphys.2014.00204 24904433 PMC4035560

[B12] Perry B. G. Cotter J. D. Mejuto G. Mundel T. Lucas S. J. (2014). Cerebral hemodynamics during graded Valsalva maneuvers. Front. Physiol. 5:349. 10.3389/fphys.2014.00349 25309449 PMC4163977

[B13] Rickards C. A. Tzeng Y. C. (2014). Arterial pressure and cerebral blood flow variability: friend or foe? A review. Front. Physiol. 5:120. 10.3389/fphys.2014.00120 24778619 PMC3985018

[B14] Rokamp K. Z. Olesen N. D. Larsson H. B. Hansen A. E. Seifert T. Nielsen H. B.. (2014b). Glycopyrrolate does not influence the visual or motor-induced increase in regional cerebral perfusion. Front. Physiol. 5:45. 10.3389/fphys.2014.00045 24575051 PMC3920105

[B15] Rokamp K. Z. Secher N. H. Eiberg J. Lonn L. Nielsen H. B. (2014a). O2 supplementation to secure the near-infrared spectroscopy determined brain and muscle oxygenation in vascular surgical patients: a presentation of 100 cases. Front. Physiol. 5:66. 10.3389/fphys.2014.00066 24611051 PMC3933814

[B16] Sørensen H. Grocott H. P. Niemann M. Rasmussen A. Hillingso J. G. Frederiksen H. J.. (2014). Ventilatory strategy during liver transplantation: implications for near-infrared spectroscopy-determined frontal lobe oxygenation. Front. Physiol. 5:321. 10.3389/fphys.2014.00321 25202281 PMC4142416

[B17] Sørensen H. Secher N. H. Rasmussen P. (2013). A note on arterial to venous oxygen saturation as reference for NIRS-determined frontal lobe oxygen saturation in healthy humans. Front. Physiol. 4:403. 10.3389/fphys.2013.00403 24478709 PMC3897873

[B18] Tarumi T. Zhang R. (2014). Cerebral hemodynamics of the aging brain: risk of Alzheimer disease and benefit of aerobic exercise. Front. Physiol. 5:6. 10.3389/fphys.2014.00006 24478719 PMC3896879

